# An integrated dataset of spatiotemporal and event data in elite soccer

**DOI:** 10.1038/s41597-025-04505-y

**Published:** 2025-02-01

**Authors:** Manuel Bassek, Robert Rein, Hendrik Weber, Daniel Memmert

**Affiliations:** 1 Institute of Exercise Training and Sport Informatics, German Sport University Cologne, Cologne, Germany; 2 DFL, German Football League, Frankfurt, Germany

**Keywords:** Research data, Education

## Abstract

Data-driven match analysis in soccer is a growing discipline in both research and practice. However, public data is scarce, which raises the barrier for entering this field and decreases reproducibility of methods and results. To bridge this gap, this paper presents a dataset of official match information, event, and position data from seven matches of the German Bundesliga’s first and second division. The match information contains meta data about the matches and their participants. The event data contain timestamps along with descriptions of discrete events, like passes, shots, or fouls. The position data contain the x/y-coordinates of every player and the ball. By integrating multiple data modalities – i.e., event logs with timestamps, and x-y coordinates of player and ball positions — the dataset offers a multidimensional view of match dynamics. This dataset supports the validation of existing analytical techniques and facilitates the development of new methodologies in sports analytics. With availability under CC-BY 4.0, it promotes transparency, reproducibility, and the idea of open science in match analysis research.

## Background & Summary

Performance analysis in team sports has become a data driven discipline in recent years^1–3^. For example, in professional soccer, game-related data are recorded using various methods and techniques for every single match across all professional leagues. These data have led to various advancements with respect to research insights and practices^1,4^. In general, three main data modalities are available: (i) Video data either from a broadcast streams or other dedicated camera systems that are used to capture, for example, the tactical behavior on the pitch (wide-angle scouting feed); (ii) event data using timestamps and descriptions of discrete match events like goals, passes and fouls; (iii) position data represented by x/y-pitch coordinates of the players and balls with a high spatial and temporal resolution. Position data have been used, for example, to characterize physiological demands of the players^5,6^, classify and evaluate tactical team behavior^7–10^, value individual actions^11,12^, or predict future states of the match^13–15^. However, these various data are usually not shared publicly due to their proprietary nature, their financial value and to maintain a competitive edge over competitors.

To date, several datasets of different modalities are publicly available. Video datasets like the *SoccerNet* dataset contains video data from multiple elite soccer matches (e.g., top-5 European leagues, FIFA World Cup). Research projects and challenges within the computer vision community as well as quality controlled manual annotations have enriched these datasets with additional information, like camera shot detection, action spotting, player identification and tracking, or pose estimation^16–19^. The event dataset *Wyscout soccer match event dataset* published by Papalardo *et al*.^20^ contains one season in five national soccer competitions and two international tournaments with detailed descriptions about on-ball actions. Similarly, the *StatsBomb Open Data* repository^21^ contains event data from various competitions and is constantly updated by the commercial event data provider Hudl StatsBomb (Agile Sports Technologies Inc., Lincoln, Nebraska, USA). Position data which, arguably, contain the most information are also the scarcest ones in volume. Although extracted position data from the *SoccerNet* dataset is available^19^, reproducing and extending this data requires expertise in computer vision and substantial computing resources^22^. To the best of our knowledge, besides such data, only few public datasets of raw position data in soccer exist with one^23^, three^24^ and nine^25^ matches, respectively. The first dataset contains of one match tracked with an optical tracking system accompanied with physical (distance covered, distances in speed zones) and tactical (team centroid position) performance indicators^23^. The second dataset used a combination of body sensors and video data to generate the position data^24^. The third dataset, has been generated by the commercial provider SkillCorner (Paris) by extracting position data from broadcast videos. Thus, player information may be limited by their visibility. Finally, Biermann *et al*.^26^ published a dataset of videos, position data, and manual annotations for 125 minutes of elite team handball.

Although common practice in other scientific fields, in particular in computer science, researchers in sports sciences and match analysis rarely publish their results on publicly available benchmark datasets^27^. This may be in fact, due to the limited availability of public data sources. Additionally, the field of match analysis is currently not open for newcomers outside of clubs as they do not have access to data. Therefore, the dataset presented in this study aims at closing this gap by publishing official, synchronized event and position data from seven German Bundesliga matches. This dataset differs from the aforementioned datasets in three ways: (i) the dataset contains the official data provided by the German Football League (Deutsche Fußball Liga; DFL)^28^ which is shared with the clubs and media instead of data generated by research projects or third party data provider; (ii) the data contains a continuous stream of all players’ and the ball’s position data, generated by a validated multi-camera tracking system; and (iii) contains both detailed event and tracking data from the same soccer matches.

Although the volume of the dataset may not allow for generalizable insights in the match analysis research domain, like several seasons of event data, and does not contain video footage of the matches, it serves as a complementary resource to be used for entry-level analyses as well as a high-quality benchmark dataset. It may therefore benefit the community by increasing the reproducibility of research from various disciplines and promoting open science practices in the field of match analysis.

## Methods

### Match information

Match information data were collected by Sportec Solutions (Sportec Solutions AG, Unterföhringen, Germany). They are derived from various data sources (clubs, weather stations, ticket sales, on-site operators) and stored and distributed from the official DFL database^28^. Consent to publish this data is given mandatorily during the player registration process. All data was provided by the original data collector, i.e., DFL with the permission to publish them under CC-BY 4.0. General information about the competitors (player’s names, jersey numbers, etc.) are entered by the respective clubs. Information about spectator numbers are based on the club’s communication and validated with ticket sale numbers. Information about the weather conditions are collected by an on-site operator from local weather stations. An operator also specifies the team tactical formation from 41 possible formation templates. If none of the templates apply to the actual formation, he is instructed to select the most similar template.

### Event data

A pool of 120 operators (trained human analysts) analyze over 616 matches every year, whereby five operators analyze each match. Operators undergo specific training for several days and are required to analyze several matches in an offline modus before their first live match. One on-site operator (speaker) and one operator in the operations center (writer) generate the initial event data stream aiming for minimum latency. Two additional operators follow the live video footage, add additional data, and check for event data quality. Each designated event is annotated in a custom software. Each event is created with a local UTC timestamp and a custom ID. Depending on the event, additional attributes, like participating players or location is added. Finally, these data are controlled for quality by a supervisor, who is a domain expert with previous one-week training and trial period.

### Position data

**Fig. 1:**
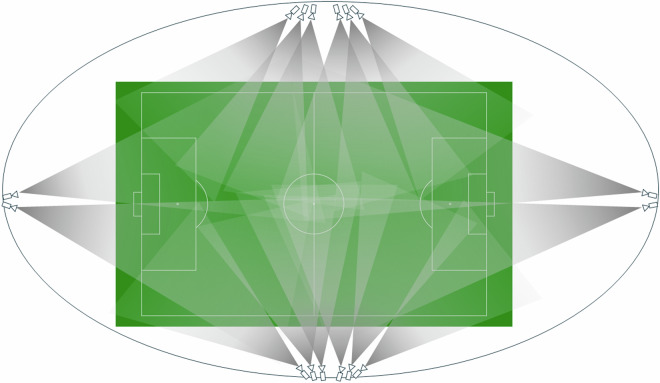
The spatial distribution of the TRACAB Gen 5 cameras following Linke *et al*.^29^.

The position data was collected using the multi-camera tracking system TRACAB Gen5 by Chyron Hego (ChyronHego Corporation, Melville, New York, USA). The camera setup has been described in detail in a recent validation study^29^ (see Technical Validation). Unlike Electronic Performance Tracking Systems, like Gobal Positioning Systems (GPS) or Local Positioning Systems (LPS), TRACAB does not rely on sensors worn by the athletes, but estimates position data from video data. Depending on the stadium, the system uses 16 to 20 cameras ($1920\times 1080$ pixels; 25 Hz). Six cameras each are positioned on both sides of the pitch and two cameras are positioned behind each goal. Figure 1 shows the general setup and camera coverage (for 16 cameras).

All moving objects on the pitch are tracked using specialized computer vision algorithms for playfield detection, player and ball detection, and player labelling^30^. The players and the ball are then projected into a local 2D coordinate system via direct linear transformation using a calibrated homography matrix. Further post-processing algorithms are applied to filter outliers and smooth the raw data.

## Data Records

The present data set is released under a CC-BY 4.0 license with the authorization from the DFL and available at figshare^31^. The dataset can also be accessed via the software package *floodlight* for the Python programming language^32^. The dataset contains data from two matches of the German Bundesliga 2022/23 season, as well as five matches from the second division Bundesliga 2022/23 season (Table 1). The Bundesliga is a top-5 league according to the UEFA country coefficient^33^. For each match, three files are available: a match information file, a match events file, and a match position data file. All three files use an XML format as the file architecture. In total, the dataset contains information about 207 players from 10 teams, 11,137 events, and 1,002,644 frames of x/y-coordinates for players and the ball. All data categories are defined in in the Catalogue of Definitions published by the DFL^34^. The video footage of the matches is not available in this dataset due to licensing restrictions.

**Table 1:** Meta data for all seven available matches.

Match-ID	Div	Home	Away	Result	xG	Date	N_Frames_	N_Events_
J03WMX	1^st^	1. FC Köln	FC Bayern München	1:2	1.33:1.18	2023/05/27	145,967	1,840
J03WN1	1^st^	VfL Bochum 1848	Bayer 04 Leverkusen	3:0	1.97:0.78	2023/05/27	141,561	1,459
J03WPY	2^nd^	Fortuna Düsseldorf	1. FC Nürnberg	0:1	1.01:1.78	2022/10/15	146,211	1,579
J03WOH	2^nd^	Fortuna Düsseldorf	SSV Jahn Regensburg	4:0	2.66:0.52	2022/08/26	137,214	1,457
J03WQQ	2^nd^	Fortuna Düsseldorf	FC St. Pauli	1:0	1.25:1.09	2022/11/05	142,345	1,679
J03WOY	2^nd^	Fortuna Düsseldorf	F.C. Hansa Rostock	3:1	2.14:0.43	2022/09/10	142,536	1,578
J03WR9	2^nd^	Fortuna Düsseldorf	1. FC Kaiserslautern	1:2	1.16:1.31	2022/11/11	146,810	1,545

N_Frames/Events_: Number of frames and events in the respective match.

### Match information

The match information files use an XML container (Box 1) and specify general meta data about the competition, the environmental conditions and competitors.

#### General

General information includes the type of sport and competition, league, season, matchday, kick-off time (ISO 8601 format), and home and away team names and IDs.

#### Environmental

Environmental conditions include the country, stadium name, ID, address, capacity, pitch dimensions (in m), weather conditions (temperature in °C, humidity in %, atmospheric pressure in hPa), and number of spectators.

#### Teams

**Fig. 2:**
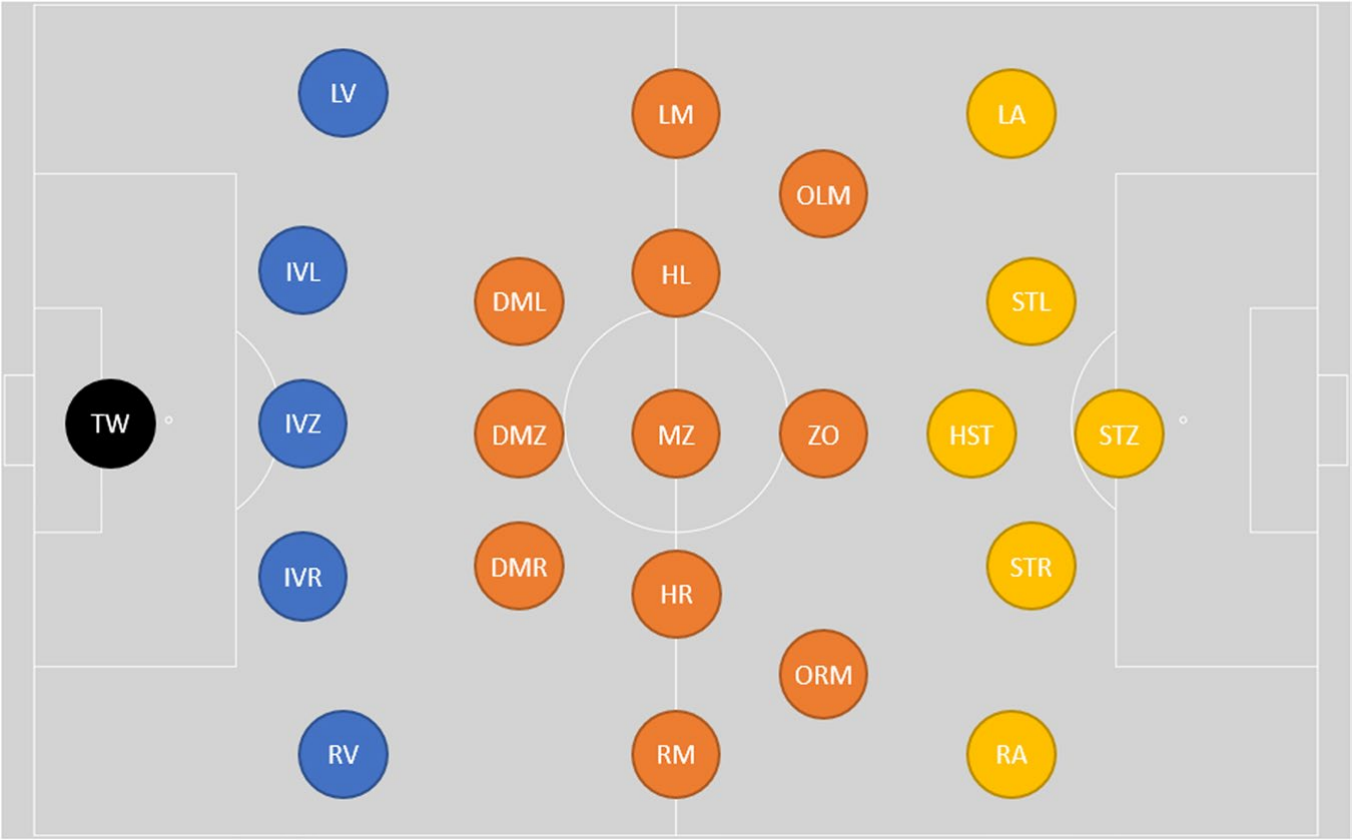
Classification of player roles in playing direction from left to right, adapted from^52^.

Team information files contain the competing teams’ IDs, names, them playing home or away, jersey colors (in hexadecimal) and tactical formation (e.g. “4-2-3-1”). Additionally, each player is listed with his ID, name, shirt number, playing position in German abbreviation (e.g., “LV” for Linksverteidiger, i.e., left back; TW for Torwart, i.e., goal keeper). Figure 2 shows the position abbreviations of players on the pitch. The starting players and team captain are listed as “true” or “false”. The coaching staff is listed with their IDs, names, and role (e.g., “trainer”; “assistantTrainer”). Similarly, the official staff is listed (e.g., “doctor”, “teamManager”).

#### Referees

The referees are listed with their IDs, names and role (e.g., “referee”; “firstAssistant”; “fourthOfficial”).

#### OtherGameInformation

This contains the gross and net playing time for the first and second half in seconds.

Box 1 Exemplary excerpt from the match information file of match J03WMX. In order to make the layout appropriate, all but two players and staff have been removed .
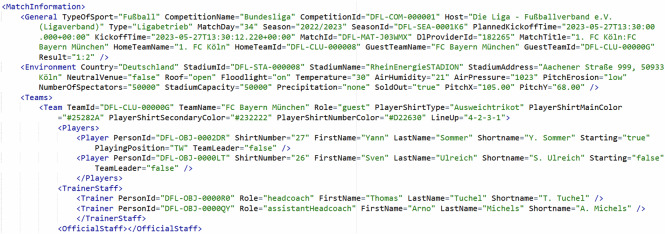

### Event data

The event data files (Box 2) contain information about discrete events categorized into player, team and referee actions. Events where one or more players participate are considered as player actions. These events include on-ball actions, tackles, fouls, off-side, and other player actions (i.e., actions that cannot be classified into specific player actions). All set pieces (e.g., kick-off, throw-in, corner, free kick) as well as counter attacks are considered team actions. Events that involve a referee decision are categorized as referee actions (e.g., start and final whistle, substitutions, sanctions).

The events are structured hierarchically (Table 2). Events are derived from general parent classes (e.g., player actions) and further specialized (e.g., on-ball action, pass). In that process, each subclass of events (e.g., blocked shot, saved shot, successful shot) inherits their parent (shot, on-ball action, player action) class’s characteristics. Each event also gets contextualized with attributes (e.g., x/y-coordinates in m, goal expectancy). All events contain the attribute timestamp which specifies the time instant when the event occurred (in ISO 8601 format).

**Table 2:** Most frequent events, their parent- and sub-classes, and attributes.

Event type	Parent-classes	Sub-classes	Attributes
Play	ThrowIn, FreeKick, Kickoff, CornerKick, GoalKick	Pass, Cross	SemiField, Player, Team, FromOpenPlay, PenaltyBox, FlatCross, Height, Distance, PlayOrigin, PlayAngle, Recipient, BallPossessionPhase, Evaluation
OtherBallAction			Player, Team, DefensiveClearance, BallPossessionPhase
TacklingGame			WinnerTeam, Winner, WinnerRole, PossessionChange, GoalKeeperInvolved, Loser, LoserRole, WinnerResult, LoserTeam, Type
Delete	*This event indicates an event deleted in post processing and does not contain information*.		
ShotAtGoal	Freekick, Penalty	ShotWide, SavedShot, BlockedShot, ShotWoodWork, SuccessfullShot, OtherShot	Team, ExtendedTypeOfShot, Pressure, GoalDistanceGoalkeeper, ShotOrigin, AssistTypeShotAtGoal, AssistShotAtGoal, AngleToGoal, PlayerSpeed, TakerBallControl, CounterAttack, ChanceEvaluation, SetupOrigin, TypeOfShot, TakerSetup, AfterFreeKick, DistanceToGoal, InsideBox, BuildUp, AssistAction, xG, BallPossessionPhase, ShotCondition
Foul			TeamFouler, Fouler, Fouled, TeamFouled, FoulType

**Fig. 3:**
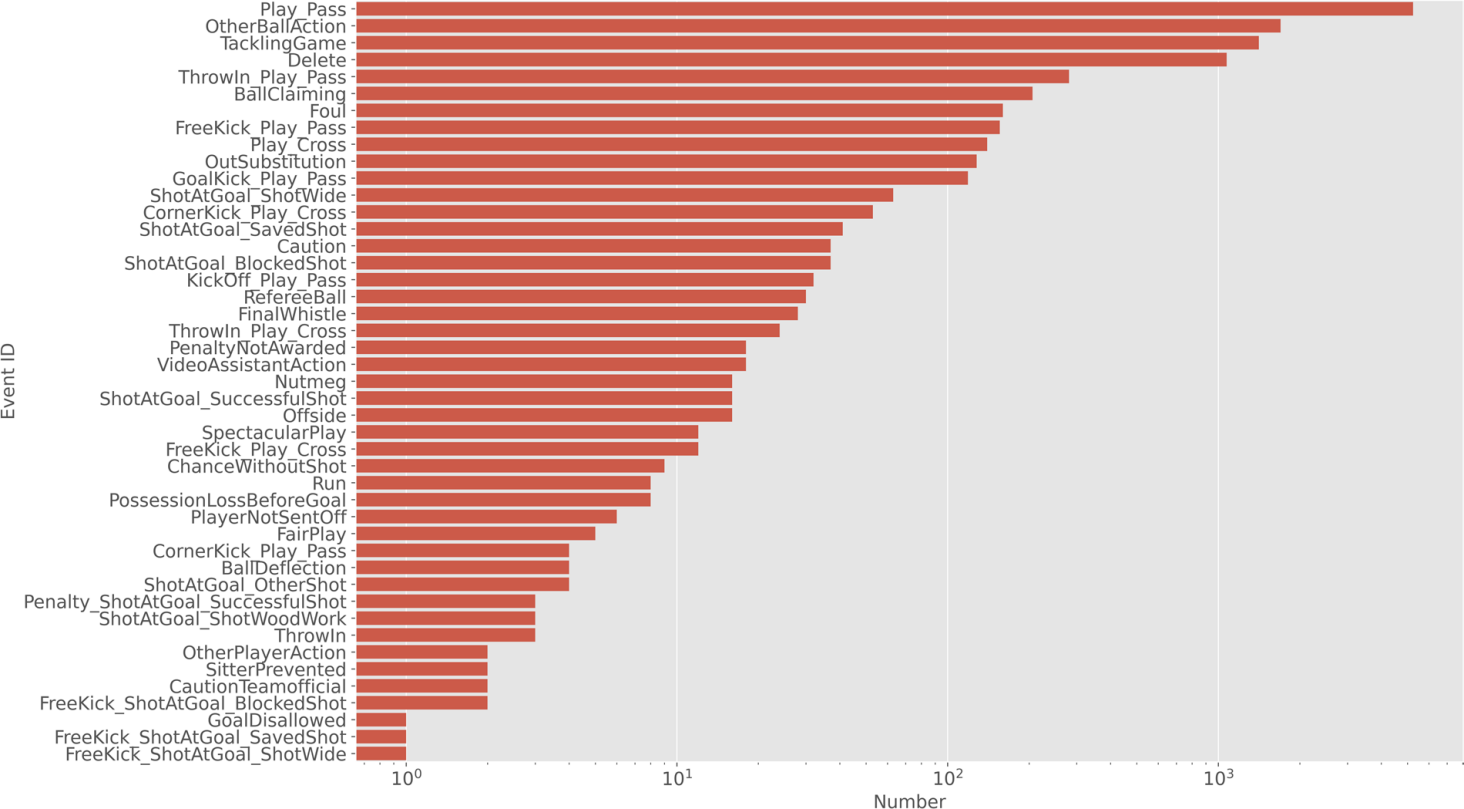
Distribution of event occurrences. The x-axis is scaled on the logarithm to base 10.

Figure 3 shows the distribution of all occurring event types (i.e., differentiated to every subclass) in the dataset. As visible, the ‘Play’ which specifies a player’s action is the most often occurring event. A ‘Play’ is an attempt from a player to switch ball control to a teammate. However, the same event class ‘Play’ can be derived from different parent classes. For instance, a free-kick is considered a team action but can be executed as a play. Also, if executed in a special way, the play can get another subclass, e.g., pass → cross. An overview of attributes for the most important events, their possible parent and subclasses, and their attributes is listed in Table 2.

The detailed list and definitions of all events and attributes is available in the Catalogue of Definitions Official Match Data^34^.

The goal expectancy (xG) is attributed to each shot at goal. Goals are in soccer are relatively rare and often influenced by randomness^35^. They may therefore not reflect the actual offensive performance of a team. The xG value estimates the probability of each shot to get converted into a goal^11^. Given a large number of observed shots as training data, shots from similar situations can be cumulated into bins and the xG value calculated as the bin’s conversion rate. The sum of xG of all shots, can be interpreted as a more accurate approximation of the offensive performance. The xG-model used in this dataset adjusts the xG value based on ten features: (i) the shot location, (ii) the speed of the player taking the shot, (iii) number of defenders in the line of the shot, (iv) goalkeepers position, (v) a “pressure”-metric^36^ on the player taking the shot, (vi) the body part, (vii) the amount of ball control prior to the shot, (viii) the amount of ball control when taking ball possession, (ix) whether the shot followed a free kick, and (x) whether the shot was a free kick^11^. The model has been trained on data from 105,627 shots taken in the German Bundesliga. An evaluation of feature importance using Shapely values shows that the features distance to goal, distance of the goalkeeper to goal and the angle to goal have the highest impact on the model output.

Box 2 The meta data and first five events from the match events file of match J03WMX .
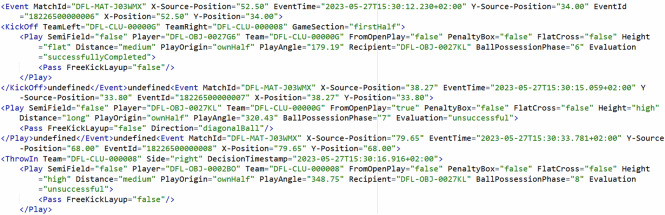

### Position data

The position data files (Box 3) specify the raw positions of each player and the ball together with further meta data. The meta data contain the respective match ID, pitch size and the start time of the data collection, i.e., kickoff of first half. For each player and the ball, the positions are stored in a list of frames for each game section (first/second half) and player. Each frame has the attributes frame number ($N$, local time stamp ($T$ in ISO 8601), x- and y-coordinates ($X/Y$ in m), distance covered since the preceding frame ($D$ in cm), speed ($S$ in km/h), acceleration ($A$ in m/s²), and minute of play ($M$).

For the ball, in addition to the position data further information about the ball height ($Z$), the ball possession status (BallPossession; 1 = home team in possession, 2 = away team in possession) and the match state (BallStatus; 0 = ball inactive, 1 = ball active) are provided. The ball possession is defined by the player in control of the ball. The match state is inactive when the match is interrupted by the referee, for example after a foul or during a substitution

Box 3 The meta data and first eight frames from the ball data in the position data file of match J03WMX .
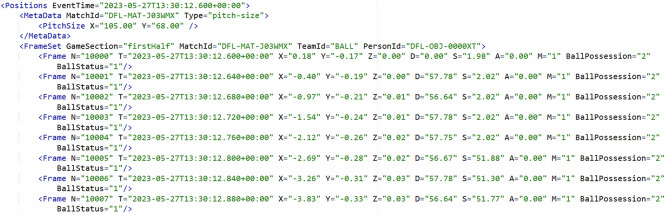

## Technical Validation

### Match information

**Fig. 4:**
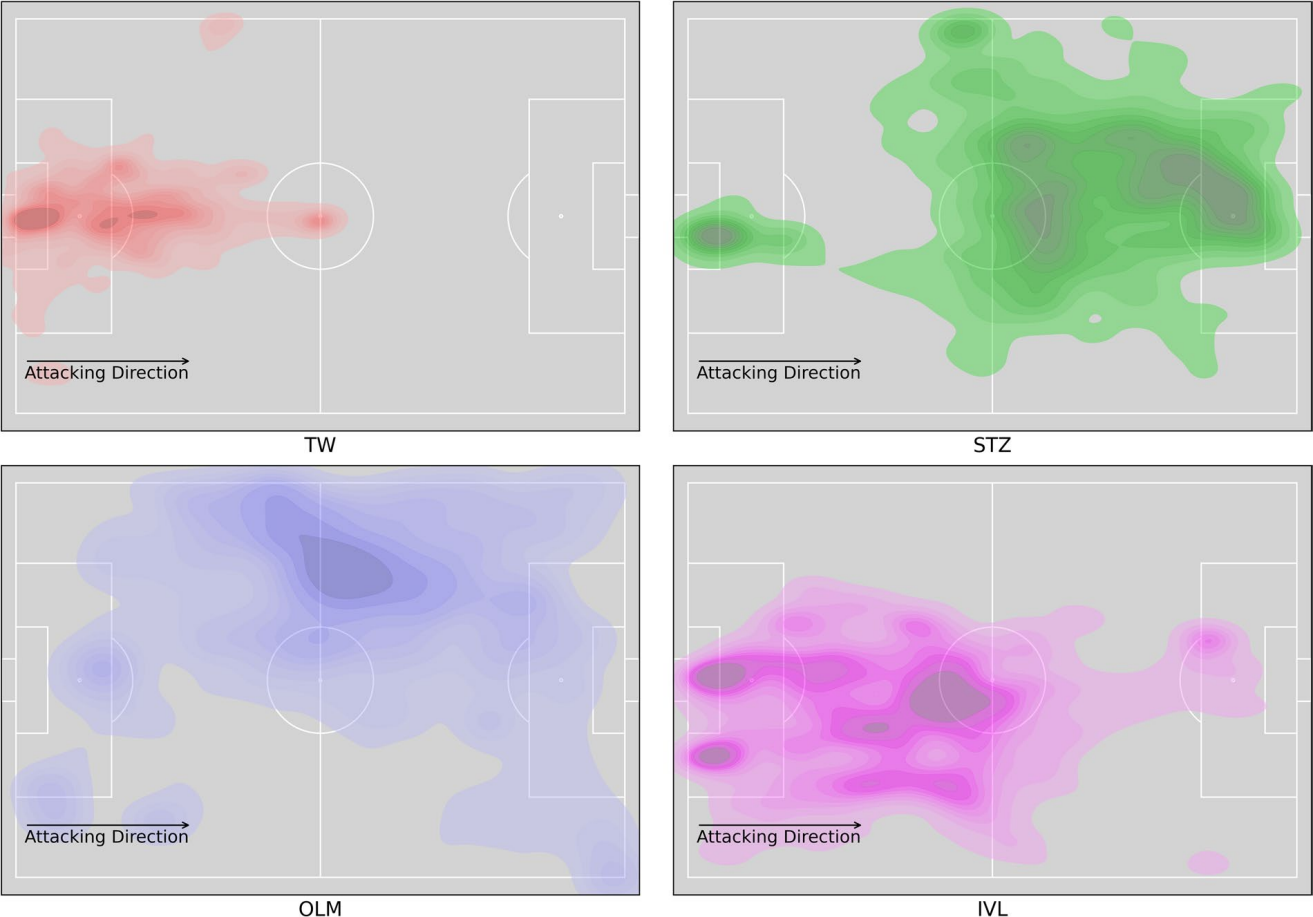
Kernel-density plot of different playing positions from the home team in match J03WOH.

The match information is extracted from the official DFL database. All data are validated throughout the process of data collection, e.g. with data from local weather stations, ticket sales records, and quality control by independent raters. To assure completeness of this dataset, all information was manually validated against other public data sources (e.g., Transfermarkt.de). The validity of the players positions, which result in the selected tactical formation is depicted in Fig. 4. The figure shows the kernel-density of players with different positions from the home team in the first half of match J03WOH (Table 1). Their general space occupation matches their position in the team formation.

### Event data

The possible errors of event tagging can be divided into semantic^26^ and temporal^37^. Semantic errors refer to false information attributed to the event. For example, a pass is labelled as successful, although in reality it failed. In this dataset, all information is checked for plausibility live and after the match to guarantee high semantic quality of the labels.

**Fig. 5:**
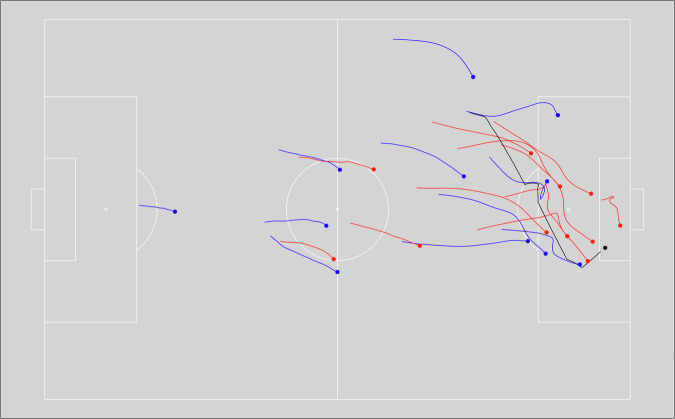
Trajectories of players for the five seconds until the first successful shot on target. Blue: Attacking team; Red: Defending team; Black: Ball; ∼: coordinates before shot; •: coordinate at the timestamp of the shot.

Temporal errors refer to inaccurate timestamps. As the event data and the position data are generated through separate processes inaccuracy may occur. This often leads to further problems downstream when analyzing the data, for example when analysts want to synchronize the event data with position data^11^. Some automatic approaches have been proposed in the literature^11,37^. However, these have not been applied to this dataset. Still, the quality of the timestamps is controlled after the game is finished. To illustrate the temporal accuracy, Fig. 5 shows the trajectories of all players and the ball five seconds before and at an exemplary time frame, the first successful shot on target in match J03WOH (Table 1).

### Expected goals

**Fig. 6:**
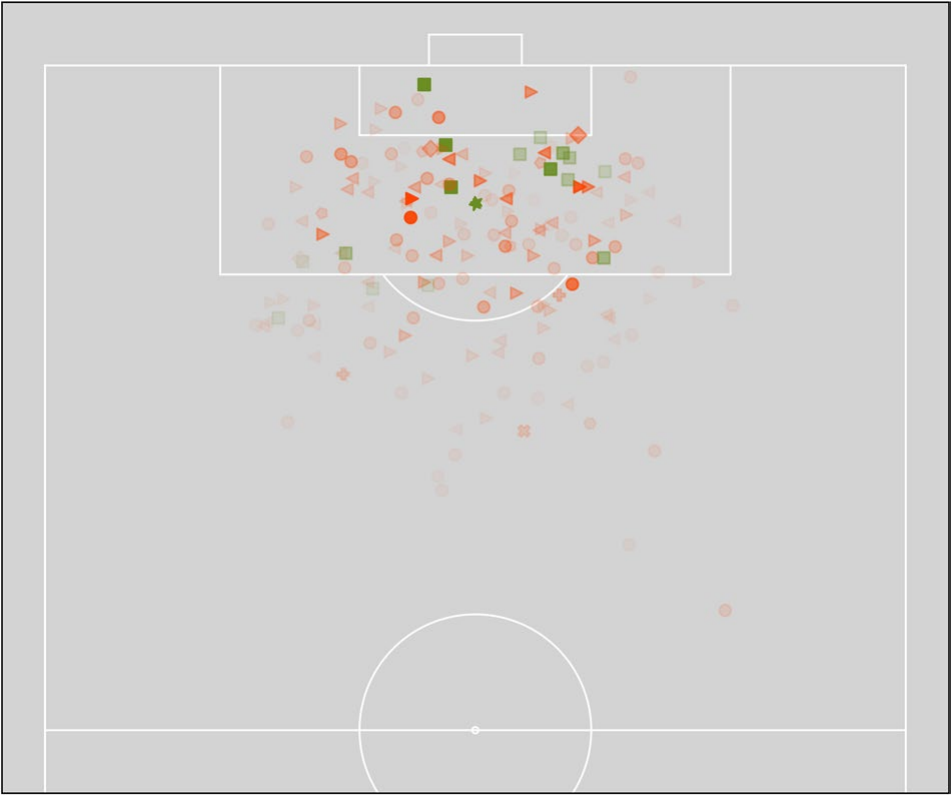
Expected goals analysis. Each marker corresponds to a shot event at this location: Green = successful shot; Red = Unsucessfull shot. Marker shape corresponds to the shot type: o = ShotWide; > = SavedShot; < = BlockedShot; ◽ = SuccessfulShot; ⬠ = OtherShot; * = Penalty; ◊ = ShotWoodWork; +  = FreeKick_BlockedShot; X = FreeKick_SavedShot; ⬡, FreeKick_ShotWide. Higher xG values correspond to lower transparency.

The dataset contains a total of 171 shots with an xG attribute. In total, 19 goals were scored in all seven matches with sum of the xG of 18.42. The xG range between 0.01 and 0.85 with an average xG of 0.11 ± 0.15, similar to the average shot-to-goal conversion rate^38^. However, the median xG is 0.05, indicating that more shots are taken at lower xG values. Figure 6 presents all shot locations, categorized by the shot type, their success and xG. Generally, most shots were performed from inside the box, and most goals were scored from inside the box. Although this small sample analysis already indicates patterns in scoring behavior and the xG only slightly underestimates the actual goals scored, the number of matches in this dataset are not sufficient to representatively evaluate or compare different xG models. However, novel models can be evaluated on this dataset to guarantee transparency on the used methods and their output.

### Position data

Generally, position data from optical tracking systems are prone to three errors: (i) inaccurate projection of the coordinates, (ii) missing data due to occlusions, (iii) false assignment of players identities (ID swaps)^30^.

Erroneous projection may happen due to issues in camera settings, pitch detection, player detection^30^, or external factors, like smoke from pyrotechnics. The resulting error has been investigated in a recent validation study^29^. An infrared camera-based motion capture system VICON (Vicon Motion Systems Ltd., Oxford, UK) was used as the reference system as it is usually referred to as the gold standard for optical tracking systems. Both systems were set up to cover a $30\times 30$ m area inside a soccer stadium. Inside the area, a soccer-specific parkour was designed to test typical movements, including accelerations, decelerations, sprints, changes of directions, and curved running paths. Additionally, a small-sided game mode was played by ten athletes. The root mean square error (RMSE) was calculated for the raw positions, velocities and acceleration data. Results showed a RSME range from 0.06 to 0.18 m, 0.03 to 0.09 m·s^−1^, and 0.06 to 0.27 m·s^−2^ for positions, velocities, and accelerations, respectively. It should be noted that the tracking error of TRACAB is greater when players are running at greater velocities. This heteroscedastic distribution of errors can also be observed for sensor-based electronic player tracking systems (EPTS)^39,40^ systems. Therefore, appropriate data cleaning algorithms, like low-pass filtering should be applied before further analysis, especially when deriving velocities and accelerations in high-intensity situations^41^.

Missing data usually occur due to occlusion of athletes during situations of high spatial density (e.g., corners)^29,30^. To resolve occlusions, the combination of multiple camera angles is necessary. The TRACAB Gen5 system uses 16 cameras and covers each area from multiple angles. During their validation study, Linke *et al*.^29^ found no erroneous or missing frames. Further, all data are visually controlled by live operators for plausibility and undergo a final quality check after the game.

The player identities are assigned manually before the start of data collection. Swaps of identities may happen if two players are in close proximity to each other and the system confuses their identity. Such swaps are resolved by the live operators or during the final quality control.

### Synchronicity between event and position data

Both, the position data and the events contain an ISO 8601 timestamp that can be used to map events onto the position data and vice versa. However, due to the human error in the manual annotation process of the event data, unsystematic errors arise when simply aligning the respective timestamps. This problem is well known in the community and has been addressed by several synchronization approaches^11,37,42^. Common to these approaches is the definition of cost functions which describe specific features for a given event. For example, at the instance of a passing event, the distance between the ball and the passing player is small and subsequently increases, accompanied by an acceleration peak of the ball. The cost functions are aggregated in a reasonable time window around the timestamp attributed to the event and the position data frame with the maximum value gets synchronized with the event. Since this approach can unintentionally swap the order of events, Kwiatkowski and Clark^43^ further suggested to use the Needleman-Wunsch algorithm^44^ to synchronize the position data and event timeseries. Van Roy and colleagues^42^ evaluated this approach und suggested a certainty value based on the unweighted sum of the cost functions.

We applied this approach using the software package *DataBallPy* (v0.5.3) for Python to synchronize all (n = 6251) passes and shots in this dataset. For the cost functions, a time difference between the event timestamp and the position data timestamp was designed that converges to one, outside of a window of ten seconds. Further, the distance between the coordinates of the event data and the ball’s position data, the distance between the player involved in the event and the ball, the rate of change in distance between the player and the ball, and the ball acceleration are modeled as sigmoid functions.

**Fig. 7:**
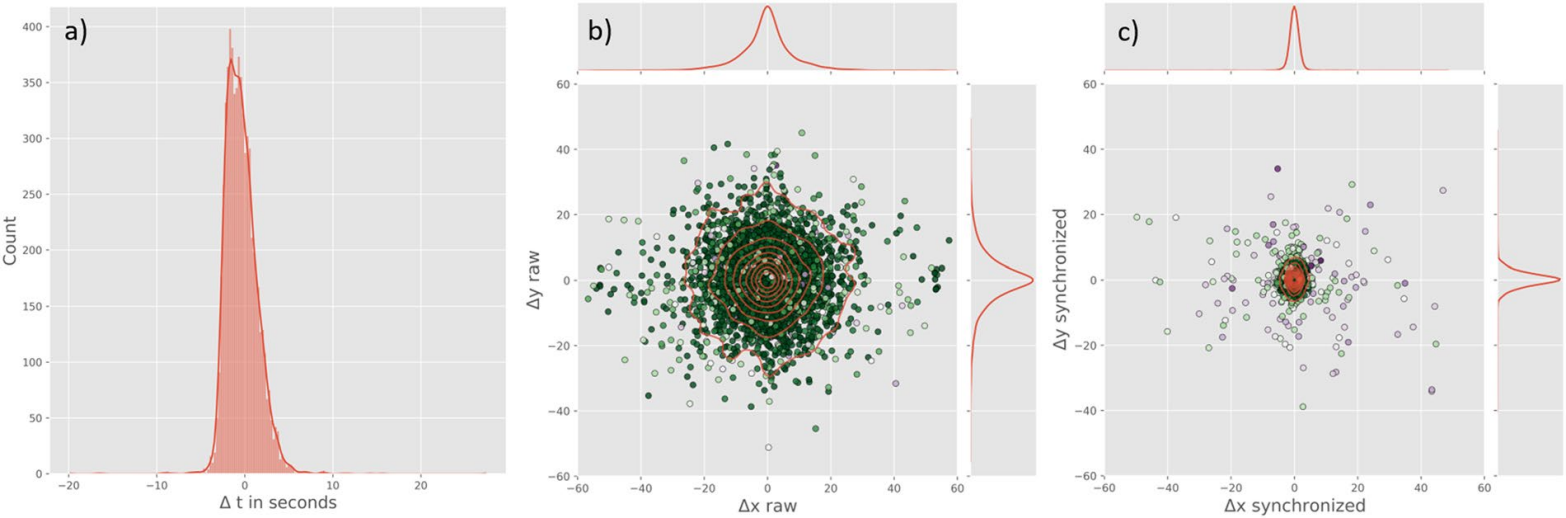
Evaluation of the event-sychronization. (**a**) Histogram of the differences between the event timestamp before and after the synchronization; (**b**) Jointplot of differences between the coordinates attributed in the event data and the ball position at the event timestamp before the synchronization; (**c**) Jointplot of differences between the coordinates attributed in the event data and the ball position at the event timestamp after the synchronization. A kernel density estimation is visible as red lines. The certainty score is color-coded for each observation with dark green, white, and purple corresponding to high, moderate, and low values, respectively.

An evaluation of the synchronization is visible in Fig. 7. The difference between the unsynchronized and synchronized timestamps is normally distributed around an average of −0.37 ± 1.82 s. The maximum difference between the unsynchronized and synchronized timestamp is 27.34 s. Similarly, the spatial difference between the coordinates in the event data and the ball coordinates in the position data at the time of the event were evaluated (Fig. 7). The differences are normally distributed around zero for both, the unsynchronized and synchronized event data. The average distance of the unsynchronized is 9.37 ± 8.39 m with a maximum distance of 58.61 m. The synchronization reduced the distance to 2.61 ± 3.60 m with a maximum distance of 75.35 m. Generally, events with high distance may be explainable by misclassifications through edge cases in the cost functions, or erroneous information in the event data (e.g., the wrong player attributed with a pass). However, as this analysis did not consider a “ground-truth” by analyzing the video footage of the matches, the underlying mechanisms are speculative. A systematic analysis of the accuracy of different synchronization algorithms would be beyond the scope for this paper, but future work can use this dataset as a benchmark for that purpose.

## Usage Notes

The *floodlight*^32^ software package (v0.4.0) for the Python programming language has been used to process and visualize the data for this paper. The package specializes in routines for parsing, processing, manipulating, and plotting data from a variety of sports data providers with a focus on scientific computing. The raw data can be downloaded from the repository and imported via the ‘floodlight.io.dfl’ module. Alternatively, it can be directly accessed via the ‘floodlight.io.datasets‘ module.

The package leverages on known packages, like *numpy*, *pandas*, or *scipy* for specialized core data objects, to represent event and position data, pitches and more. The *floodlight* package also provides out of the box methods for smoothing (e.g., Butterworth low-pass filter) calculating metrics for intensity (distance, velocity, acceleration, metabolic power), approximate entropy, geometric properties (team centroid, stretch index), space control (Voronoi tesselation), and plotting individual data points or trajectories. All implemented models have been used and evaluated in peer-reviewed publications. The package has an extensive documentation and getting-started tutorials for newcomers in the field of match analysis in sports and is suitable for professional, non-professional and teaching environments, alike (https://floodlight.readthedocs.io/).

Since this dataset is limited to seven matches, the generalizability of findings in with regard to match analysis may be low. Although approaches to match analysis considering only single matches exist^45,46^, more recent domain specific analysis contain over 100 studies on average^47,48^ with up to 1200 matches^49^. Since the outcome of single matches may be heavily influenced by individual decision making or luck^35^ robust findings in match analysis should be based on representative sample sizes^50,51^.However, the strength of this dataset is that it can be utilized for a variety of reproducibility and benchmarking tasks currently lacking in the literature, especially methodological approaches including but not limited to space control, expected value, data synchronization, trajectory prediction, network analysis, trajectory clustering, formation detection, data mining, game segmentation, and visualization^3^.

## Data Availability

The code used for the visualizations is available on GitHub (https://github.com/spoho-datascience/idsse-data). All figures have been produced with Python v3.10, *floodlight* v0.4.0, and *seaborn* v0.13.2
